# Discrete Element Simulation of the Effect of Roller-Spreading Parameters on Powder-Bed Density in Additive Manufacturing

**DOI:** 10.3390/ma13102285

**Published:** 2020-05-15

**Authors:** Jiangtao Zhang, Yuanqiang Tan, Tao Bao, Yangli Xu, Xiangwu Xiao, Shengqiang Jiang

**Affiliations:** 1 Institute of Manufacturing Engineering, Huaqiao University, Xiamen 361021, China; zhangjiangtao@stu.hqu.edu.cn (J.Z.); Baotao@stu.hqu.edu.cn (T.B.); xuyangli@hqu.edu.cn (Y.X.); 2 National & Local Joint Engineering Research Center for Intelligent Manufacturing Technology of Brittle Material Products, Huaqiao University, Xiamen 361021, China; 3 School of Mechanical Engineering, Xiangtan University, Xiangtan 411105, China; xiaoxiangwu@xtu.edu.cn (X.X.); jsqcx@xtu.edu.cn (S.J.)

**Keywords:** DEM, roller-spreading parameters, powder-bed density, additive manufacturing

## Abstract

The powder-bed with uniform and high density that determined by the spreading process parameters is the key factor for fabricating high performance parts in Additive Manufacturing (AM) process. In this work, Discrete Element Method (DEM) was deployed in order to simulate Al_2_O_3_ ceramic powder roller-spreading. The effects of roller-spreading parameters include translational velocity *V_s_*, roller’s rotational speed *ω*, roller’s diameter *D,* and powder layer thickness *H* on powder-bed density were analyzed. The results show that the increased translational velocity of roller leads to poor powder-bed density. However, the larger roller’s diameter will improve powder-bed density. Moreover, the roller’s rotational speed has little effect on powder-bed density. Layer thickness is the most significant influencing factor on powder-bed density. When layer thickness is 50 μm, most of particles are pushed out of the build platform forming a lot of voids. However, when the layer thickness is greater than 150 μm, the powder-bed becomes more uniform and denser. This work can provide a reliable basis for roller-spreading parameters optimization.

## 1. Introduction

The powder-bed based additive manufacturing (AM), such as Inkjet Three-Dimensional (3D) Printing, selective laser sintering (SLS), and selective laser melting (SLM) are the most widely used additive manufacturing technologies [1,2]. The powder-bed density directly affects the effective powder thermal conductivity; consequently, the temperature distribution in melt pool and performance of AM parts [3]. The powder-bed with uniform and high density is the key factor to fabricate high-performance parts in AM processes [3,4]. Otherwise, the loose packing of powder-bed leads to defects, such as inner holes and poor performance of AM parts.

Currently, the measurement of powder-bed density is still facing challenges. Recently, the method of measuring powder-bed density by weighing the powder inside containers was proposed [5,6]. However, the method is rather uncertain due to heavily depending on the powder spreading process. It is worth mentioning that Usman et al. [4] presented a new method for the measurement of powder-bed density. The powder samples were bonded through an ultraviolet curable polymer at different locations. The compactness density of the powder-bed will be obtained by a nano-computing tomography (CT) scanning results. Although the method is high accuracy, the cost of the experiment is too expensive to be applied in industry.

With AM technology growing rapidly in industry and academia, there has been increasing interest in the application of Discrete Element Method (DEM) in AM processes during the past five years. Zohdi [7,8] and John C. [9] proposed DEM to simulate the process of AM, which establishes a more realistic particle contact model and consider the effects of multiple physical factors on the particle system. Subsequently, much work applied DEM to simulate powder spreading process [10,11,12,13,14,15,16,17]. These investigations indicate that DEM is a helpful tool for simulating powder mechanical behavior to overcome the experimental challenges in AM processes.

There are mainly two main methods for powder spreading: blade-spreading [10,16] and roller-spreading [13,18]. In the blade-spreading process, the straight movement of the blade is simple and easy to control; however, the powder bed cannot be compacted. For the roller-spreading process, the roller movement, including translation and counter-clockwise rotation, is relatively more complex than the blade. The advantage of roller-spreading is that it can get higher powder-bed density than that of the blade-spreading [16]. However, previous works focus on metal powder spreading by blade [6,12].

Eric J.R. [13] studied the effect of spreading conditions on the powder-bed roughness in roller-spreading process and showed that at the increasing the roller’ translational speed leads to an increase in the surface roughness of the powder bed. Nan [19] studied the effects of gap height and roller’ rotational speed on the evolving particle trajectory and spread layer uniformity by DEM. The results show that the segregation extent decreases as the gap height increases or the roller’ rotational speed decreases. However, the roller-spreading parameters of the above studies are simple and roller-spreading parameters are relatively complex, which has an important influence on powder flow and powder-bed densification mechanism. Thus, further research is needed in order to elucidate on the roller-spreading parameters for improving powder-bed density and processing efficiency.

In this work, DEM is used to simulate Al_2_O_3_ ceramic powder spreading by roller in AM processes. The Hertz–Mindlin with JKR model of Al_2_O_3_ ceramic powder is established. Simultaneously, the DEM parameters are calibrated. Subsequently, powders in the powder spreading process are divided into three zones: I. Powder free-flow zone, II. Powder compression zone, and III. Powder layer zone. A mathematical-statistical method is employed to quantitatively measure the powder-bed compactness density and uniformity of powder-bed density. Finally, the effect of roller- spreading parameters on Al_2_O_3_ ceramic powder-bed density is analyzed.

## 2. Materials and Methods

### 2.1. Discrete Element Method

The DEM method with “soft ball model” was developed by Cundal and Strack [20]. It has been widely used to simulate powder behavior in AM processes [6,12]. In DEM equations of the linear motion and rotation motion of particle are governed by Newton’s second law:

(1) $$m_{i}\frac{dv_{i}}{dt}=\sum_{j}\left(F_{ij}^{n}+F_{ij}^{t}\right)+F_{i}^{g}$$

(2) $$I_{i}\frac{d\omega_{i}}{dt}=\sum_{j}\left(T_{ij}^{s}+T_{ij}^{r}\right)$$

where $m_{i}$ $I_{i}$ $v_{i}$, and $\omega_{i}$ are the mass, moment of inertia, translational velocity, and rotational velocity of particle *i*; $F_{ij}^{n}$ and $F_{ij}^{t}$ are the normal contact force and tangential contact force imposed on particle *i* by particle *j* or wall; $F_{i}^{g}$ is the gravitational force of particle *i*; and, $T_{ij}^{s}$ and $T_{ij}^{r}$ are the torques on particle *i* caused by tangential force and rolling friction.

The Hertz–Mindlin JKR model [21] is taken into account simulations to account for adhesion force, which adds an additional normal cohesive force within the contact zone:

(3) $$F_{ij,c}^{n}=\frac{4E^{*}}{3R^{*}}\alpha^{3}-4\sqrt{\pi \gamma E^{*}}\alpha^{\frac{3}{2}}$$

The normal damping force is described as:

(4) $$F_{ij,d}^{n}=-2\sqrt{\frac{5}{6}}\beta \sqrt{S_{n}m^{*}}v_{n}^{\bar{rel}}$$

where $E_{i}$, $\gamma_{i}$, $R_{i}$ and $E_{j}$, $\gamma_{j}$, $R_{j}$ are the Young’s Modulus, Poisson ratio, and radius of contact element *i* and *j*, $v_{n}^{\bar{rel}}$ is the normal relative velocity between contact element *i* and *j*, α is radius contact area. Table 1 defines the above parameters used in the contact model.

The tangential force between element *i* and *j* consists of two components: the tangential contact force and tangential damping force are defined, as following [22,23]:

(5) $$F_{ij,c}^{t}=-S_{t}\delta_{t}$$

(6) $$F_{ij,d}^{t}=-2\sqrt{\frac{5}{6}}\beta \sqrt{S_{t}m^{*}}v_{t}^{\bar{rel}}$$

The tangential force is limited by Coulomb friction according to $F_{ij,c}^{t}<\mu_{s}F_{ij}^{n}$.

The torque on particle *i* due to the tangential force is:

(7) $$T_{ij,c}^{s}=R_{i}F_{ij}^{t}$$

The torque acted on particle *i* due to the rolling friction is:

(8) $$T_{ij}^{r}=-\mu_{r}F_{ij,c}^{n}R_{i}\omega_{i}$$

where *μ_s_* and *μ_r_* are the coefficient of static and rolling friction, respectively.

The model calculates attractive cohesion forces, even when the particles are not in real contact. The critical gap between particles *δ_n_*_,*c*_ and critical contact spot radius *α_n_*_,*c*_ are given by [24]:

(9) $$\delta_{n,c}=\frac{\alpha_{c}{}^{2}}{R^{*}}-\sqrt{\frac{4\pi \gamma \alpha_{c}}{E^{*}}}$$

(10) $$\alpha_{n,c}=\frac{9\pi \gamma R^{{*}^{2}}}{2E^{*}}\left(\frac{3}{4}-\frac{\sqrt{2}}{2}\right)$$

When the particles are not in actual contact and the gap between particles is smaller than *δ_n_*_,*c*_, the adhesion reaches a maximum value, called “pull-out force”, and the maximum cohesive force *F_pullout_* is given by [24,25]:

(11) $$F_{pullout}=-\frac{3}{2}\pi \gamma R^{*}$$

### 2.2. Calibration of DEM Parameters

For the DEM simulation of the spreading process, DEM parameters, including powder material property parameters and contact model parameters, play a crucial role in getting the correct results [26,27].

**Figure 1:**
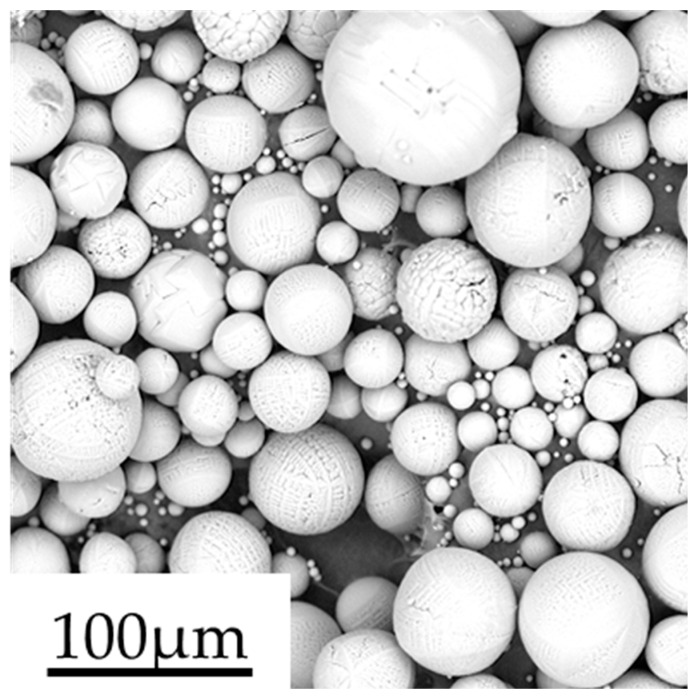
Scanning electron microscope (SEM) micrograph of Al_2_O_3_ ceramic powders.

The Al_2_O_3_ ceramic powders are widely used in AM processes [28,29,30]. In this work, Al_2_O_3_ ceramic powders (Dongchao Technologies, Dongguan, China) are the research object. Particle size, density, shear modulus, and Poisson’s ratio are the powder material property parameters. The particles size and density are measured in order to calibrate the DEM parameters. A dynamic optical particle analyzer measures the particle size (Retsch Camsizer X2, Retsch technology GmbH, Haan, Germany). The particle size D (10), D (50), and D (90) are 24, 48, and 72 μm. Particles density is measured by a Gas Pycnometer (Bei Shi De Instrument-S & T, Beijing, China) with a value of 3820 kg/m^3^. The micromorphology of powders is observed by secondary electron imaging in the scanning electron microscope (SEM, Phenom ProX, Phenom-World, Eindhoven, Holland). Figure 1 shows the SEM micrograph for Al_2_O_3_ ceramic powders around ball shape. The shear modulus and Poisson’s ratio are drawn from reference literature [31]. According to the literature [32], Young’s modulus has little effect on the simulation results. The value of Young’s modulus is set as 3 GPa, which is decreased two orders of magnitude than the actual value in order to reduce computer time consumption.

**Figure 2:**
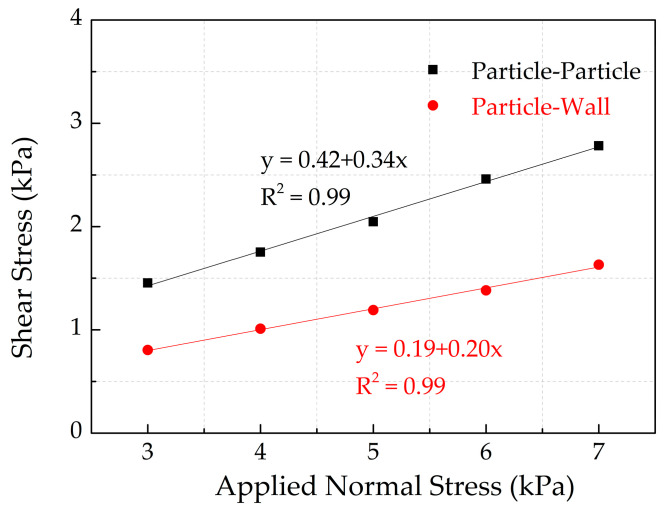
Shear test results of powder-powder and powder-wall.

**Figure 3:**
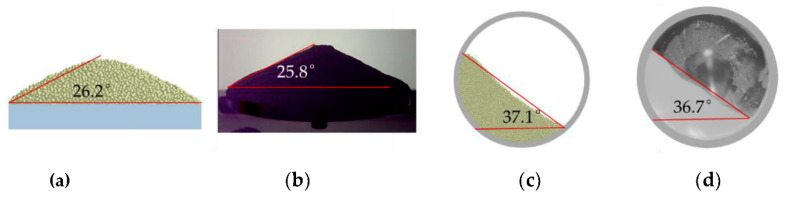
Comparing simulations and experiments: (**a**) Simulations results of angle of repose (AOR); (**b**) Experimental results of AOR; (**c**) Simulations results of avalanche angle; and, (**d**) Experimental results of avalanche angle.

The contact model parameters include the coefficient of restitution, static friction, and rolling friction. The static friction coefficients of particle-particle and particle-wall are measured by the Jenike shear test defined in ASTM D6773 [33], which has been widely used as a measure for powder flowability. Figure 2 shows the shear test results. The static friction coefficients of powder-powder and powder-wall are set as 0.34 and 0.20, respectively. It is very difficult to measure the rolling friction coefficient of fine powder. The Al_2_O_3_ ceramic is a typically hard and brittle material. The rolling resistance of Al_2_O_3_ ceramic powder is very weak. Therefore, the rolling friction coefficient is set as a small value of 0.05. The value of the restitution coefficient can reference literature [31]. According to the literature [15], the size of the DEM system has little effect on the angle of repose (AOR). The AOR defined in ISO-4490 [34] is a common technique, where powder flows through the funnel onto the plate and the slope angle of the developed cone to the base plate is the AOR and considered as a measure for powder flowability. The surface energy density *γ* of particle-particle and particle-wall is calibrated by comparing the simulations and experiments of the AOR, as shown in Figure 3a,b. Finally, simulations and experimental results of the avalanche angle fit well as shown in Figure 3c,d. Table 2 provides the DEM parameters of particles.

### 2.3. Simulation Conditions

**Figure 4:**
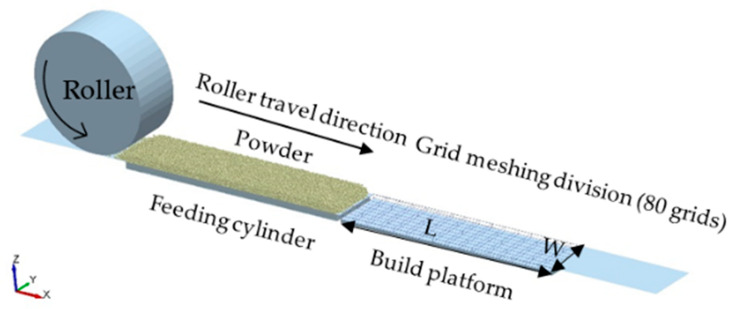
DEM simulation of roller-spreading process.

The roller-spreading simulations include a feeding cylinder and a build platform. The width *W* and length *L* of the feeding cylinder and the build platform are 2 and 10 mm, as shown in Figure 4. The roller-spreading model is established as follows: initially, 10,000 particles are generated to form the powder-bed on the top of the feeding cylinder. Subsequently, the roller moves from left to right at speed *V_s_* while it rotates counter-clockwise direction at speed *ω*. The powders are transported into the build platform forming powder layer by the roller. Table 3 shows the value of principal roller-spreading parameters. All of the simulation results presented in this work were carried out with DEM software, EDEM©.

### 2.4. Characterization of Powder-Bed Density

It is necessary to first characterize powder-bed density in order to study how roller-spreading parameters affect powder-bed density. The build platform is divided into 80 (4 × 20) grids as shown in Figure 3. The size of each grid is *L_x_* (500 μm) × *L_y_* (500 μm) × *L_z_*, where *L_z_* represents the thickness of powder layer formed in individual spreading process. We define average fraction $\bar{\varphi }$ and fraction standard deviation *S* of grids to characterize the powder-bed compactness density and the uniformity of powder-bed density, respectively. The calculation fraction of powder layer in the grid is given:

(12) $$\varphi_{j}=\frac{\sum_{i}^{n}\frac{4}{3}\pi R_{i}^{3}}{L_{x}L_{y}L_{z}}$$

where $R_{i}$ is the radius of particle *i*, *L_x_* (500 μm) × *L_y_* (500 μm) are the lateral dimensions of the grid; *n* is the total number of particles in the j-th grid.

The average fraction $\bar{\varphi }$ of grids is:

(13) $$\bar{\varphi }=\frac{\sum_{j=1}^{N}\varphi_{j}}{N}$$

where *N* is total number of grids.

The fraction standard deviation *S* of grids can be expressed as:

(14) $$S=\sqrt{\frac{1}{N-1}\sum_{i=j}^{N}{\left(\varphi_{j}-\bar{\varphi }\right)}^{2}}$$

## 3. Results and Discussion

**Figure 5:**
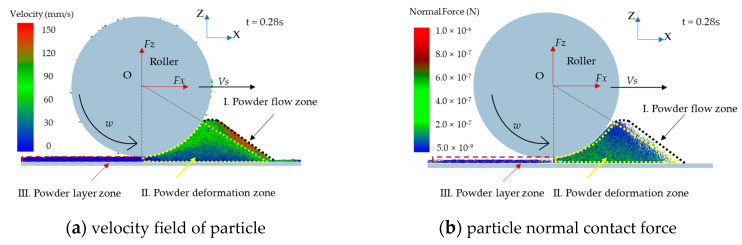
Powders are divided into three zones when *D* = 5 mm, *V_s_* = 80 mm/s, *ω* = 200 rpm, and *H* = 150 μm at t = 0.28 s.

Spreading powders are divided into three zones according to powder velocity or its normal force state, as shown in Figure 5.

(1) Powder free-flow zone: the powder flow under the action of weight force and external force exerted by roller. The normal contact forces between particles are weak, but particles velocities are relatively higher in this zone. The particles in the zone that will fall into the build platform to form powder layer.
(2) Powder compression zone: the powder-bed is compressed and deformed forming a considerably dense powder layer by roller. The particle normal contact force is strong and particles below the roller are squeezed in this zone with low velocity.
(3) Powder layer zone: powder layer underneath the surface of the roller is formed and flattened by the translation motion of the roller. The particles in this zone keep stable.

### 3.1. The Effect of Roller’s Translational Velocity on Powder-Bed Density

**Figure 6:**
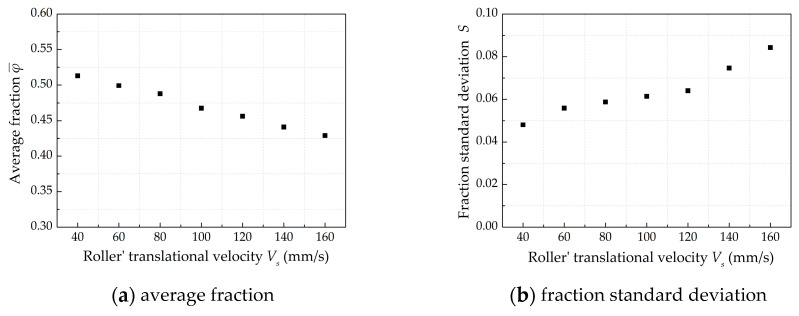
The effect of roller’s translational velocity on powder-bed density when *D* = 5 mm, *ω* = 200 rpm, *H* =150 μm.

Figure 6a shows the effect of translational velocity *V_s_* on average fraction $\bar{\varphi }$. The average fraction $\bar{\varphi }$ decreases with a linear trend from 0.51 to 0.43 with the *V_s_* increasing. The fraction standard deviation *S* increases from 0.048 to 0.084 as the translational velocity increases, and the increasing trend becomes more significant when the *V_s_* is greater than 120 mm/s, as shown in Figure 6b. The results show that the increased *V_s_* lead to a poor powder-bed compactness density and uniformity of powder-bed density.

**Figure 7:**
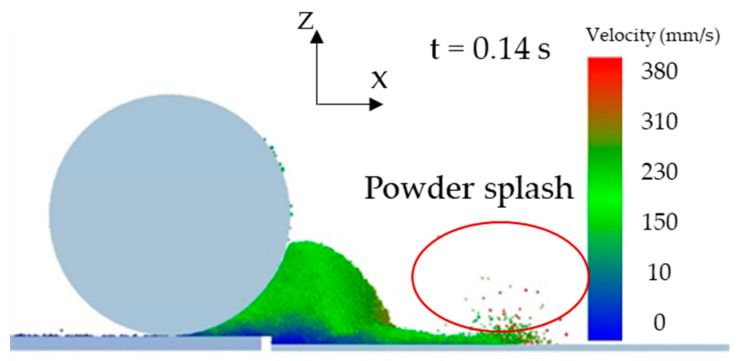
The powder flow pattern when *V_s_* is 160 mm/s at t = 0.14 s.

**Figure 8:**
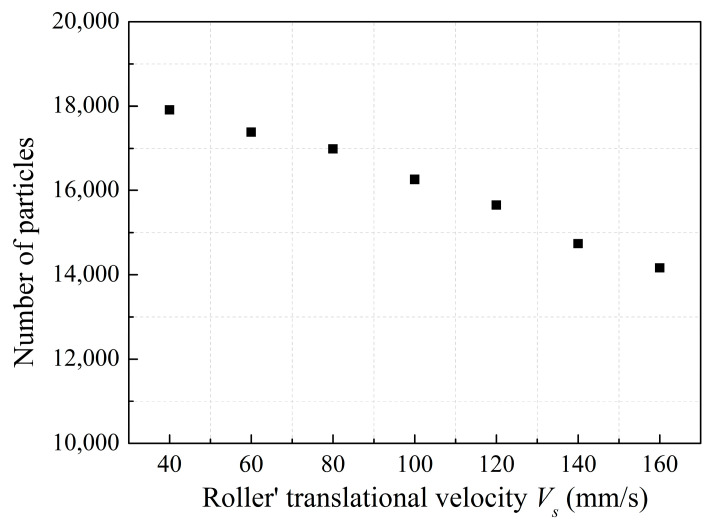
Number of particles in powder layer zone.

**Figure 9:**
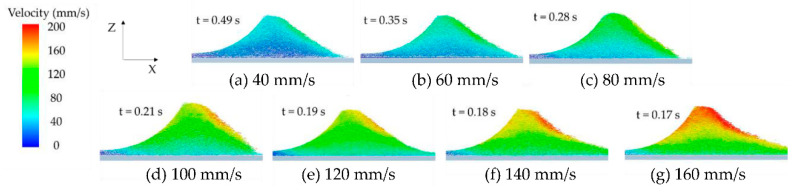
Powder flow velocity under different roller’s translational velocity.

When the roller’s translational velocity is 160 mm/s, the powder will fly up leading to particle splash, which will lead to a poor powder-bed density, as shown in Figure 7. In addition, powder splash can contaminate the key parts of the AM machine. We have performed statistical analysis on the number of particles in the powder layer zone in order to further analyze the effect of *V_s_* on the powder layer zone. Figure 8 shows that the number of particles decreases by 20% from 17,908 to 14,162 in powder layer zone when the *V_s_* increases from 40 to 160mm/s. When the *V_s_* increases, particles velocity also increases in the powder deformation zone, as shown in Figure 9. The high velocity of particles in the powder deformation zone crossing the gap between the roller and substrate will keep moving for a longer distance before residing on the substrate, leading to the number of particles decreasing in the powder layer zone [35]. Therefore, the increased *Vs* lead to a poor powder-bed density. Comparing the results of work [13], increasing the translational velocity of blade also leads to poor powder-bed quality.

According to the above results, the translation velocity should be as low as possible for improving the powder-bed density. However, this is in conflict with the production efficiency in engineering. In AM processes, the part is built up by layer-by-layer, and the translation velocity mainly determines the powder spreading time. It takes much time if the translation velocity is too low. Therefore, the minimum translational velocity should be limited to properly balance the powder-bed density and processing efficiency.

### 3.2. The Effect of Roller’s Rotational Speed on Powder-Bed Density

**Figure 10:**
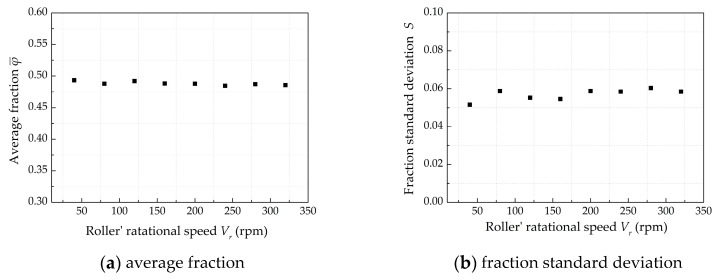
The effect of roller’ rotational speed on powder-bed density when *D* = 5 mm, *V_s_* = 80 mm/s, *H* = 150 μm.

The *ω* of roller was set from 40 to 320 rpm in order to investigate the effect of roller’s rotational speed *ω* on powder-bed density, and Figure 10 shows the effect of *ω* on powder-bed density. With the increase of *ω*, the value of $\bar{\varphi }$ fluctuates around 0.49, which shows no significant changes. It can be seen from Figure 10b that the fraction standard deviation *S* also fluctuates around 0.058. The results show that the *ω* has little effect on the powder-bed density.

**Figure 11:**
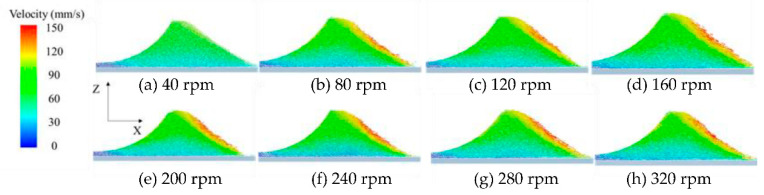
Powder flow velocity under different roller’s rotational speed at t= 0.28 s.

Figure 11 shows powder flow velocity under different roller’s rotational speed. When the particles near the roller reach the highest point under its action, particles begin to slide down entering the free-flow zone due to gravity. Particles velocity in the free-flow zone at 40 rpm is lower, while particles velocity shows no significant difference in the free-flow zone when *ω* increases from 80 to 320 rpm. In the powder deformation zone, particles velocity shows no significant difference when the *ω* increases from 40 to 320 rpm. Therefore, the *ω* has little effect on the powder-bed density. Usually, the higher particles velocity can overcome the adhesion energy of particles due to the greater kinetic energy. The powder flowability in the free-flow zone will be improved, which is easy to fall into the platform. It is known that adhesive and cohesive forces of fine powders lead to mostly unwanted agglomeration and poor powder flowability. Therefore, the higher *ω* is recommended in order to overcome poor powder flowability.

### 3.3. The Effect of Roller’s Diameter on Powder-Bed Density

**Figure 12:**
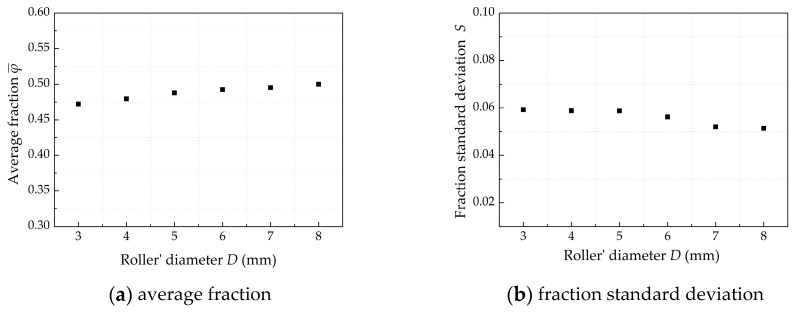
The effect of roller’s diameters on powder-bed density when *V_s_* = 80 mm/s, *ω* = 200 rpm, *H* = 150 μm.

This paper carried out DEM simulation on the roller-spreading process under different roller’s diameters in order to study the effect of roller structure parameters on the powder-bed density. It can be seen from Figure 12a that the $\bar{\varphi }$ increases from 0.47 to 0.50, when the diameter of the roller increases from 3 to 8 mm. It can be seen from Figure 12b that the fraction standard deviation *S* decreases. The results show that a larger roller’s diameter can get better powder-bed density.

**Figure 13:**
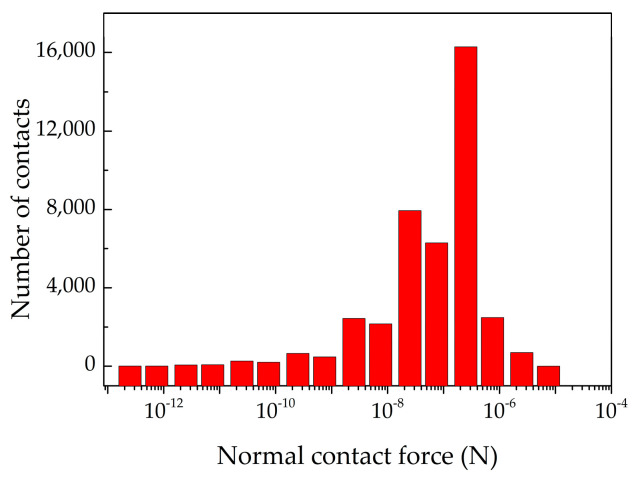
Number distribution of the normal particle contact force when *D* = 5 mm, *V_s_* = 80 mm/s, *ω* = 200 rpm, *H* = 150 μm at t = 0.28 s.

**Figure 14:**
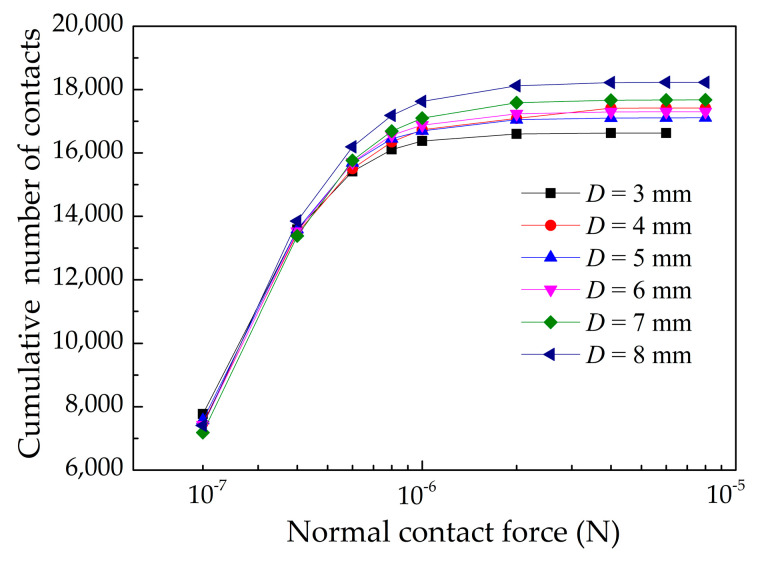
Cumulative numerical distribution of the normal particle contact force bigger than 1.0 × 10^−7^ N in the powder compression zone under different roller’s diameters when *V_s_* = 80 mm/s, *ω* = 200 rpm, *H* = 150 μm at t = 0.28 s.

Powders are divided into three zones according to powder velocity or normal contact force state, as shown in Figure 5. Figure 5b shows that the force in the particle system is disorganized. We can see that the particles are compacted by load of the roller. The force is attenuating from powder compression zone to powder free-flow zone. The particles are compressed and deformed forming a relatively dense powder layer in the powder compression zone. Therefore, the force in the powder compression zone is stronger than other zones. The number of normal contacts is quantitatively investigated in the powder free-flow zone and the powder compression zone, as shown in Figure 13. The high velocity powders with weak normal contact force in the range of 1.0 × 10^−13^ N to 1.0 × 10^−7^ N in the powder free-flow zone, as shown in Figure 5b. The strong normal contact force bigger than 1.0 × 10^−7^ N in the powder compression zone is the main driving force for the densification of the powder-bed. Figure 14 shows the cumulative number of particle normal contacts when normal contact force bigger than 1.0 × 10^−7^ N in the powder compression zone under different roller’s diameters. When *D* is 3 mm, the maximum magnitude of particle normal contact force is 6.0 × 10^−6^ N and number of particle normal contacts is 16,000. When *D* increases, the particle normal contact force is strengthening and the number of particle normal contacts is 18,000 when *D* = 8 mm. The contact zone between the roller and powder in the powder compression zone will increase. This is why increasing the roller’s diameter can lead to the densification of powder-bed.

**Figure 15:**
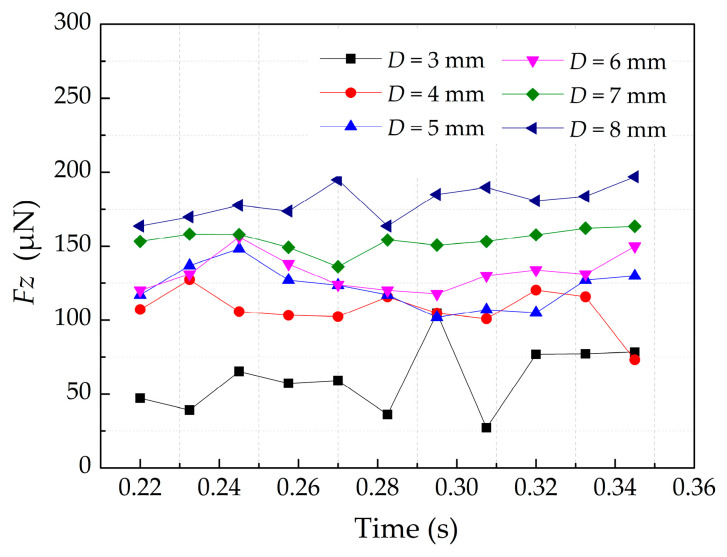
The total force of the roller in the vertical directions under different roller’s diameters.

The force of roller is discussed in the following in order to further analyze the force between the powder and roller. When the roller begins to enter the build platform, the total force of the roller in the vertical directions *F_z_* is collected as shown in Figure 15. We can see that *F_z_* show great changes when the roller moves over build platform. Obviously, the simulation results show the *F_z_* increase with the diameter of roller, which means greater pressure on powder-bed. Therefore, the diameter of roller should be increased within a certain range in order to increase the powder-bed density.

### 3.4. The Effect of Powder Layer Thickness on Powder-Bed Density

**Figure 16:**
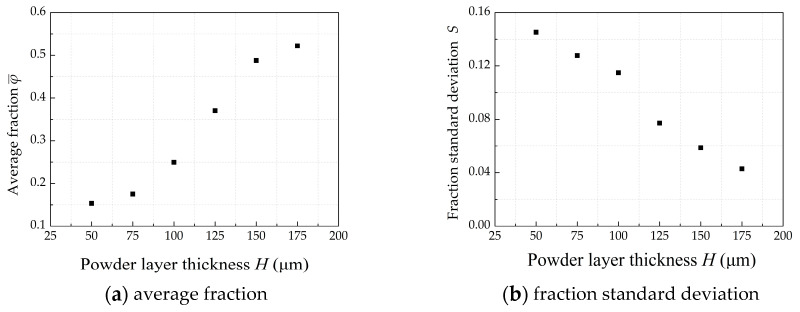
The effect of powder layer thickness on powder-bed density when *D* = 5 mm, *V_s_* = 80 mm/s, *ω* = 200 rpm.

Usually, the layer thickness of roller-spreading is from 50 to 175 μm. Figure 16a shows that the $\bar{\varphi }$ varies from 0.15 to 0.52 when the layer thickness increases from 50 to 175 μm. It can be seen from Figure 16b that the fraction standard deviation *S* is reduced as increasing the powder layer thickness. This finding suggests that the layer thickness is the most important factor that affects the powder-bed density.

**Figure 17:**
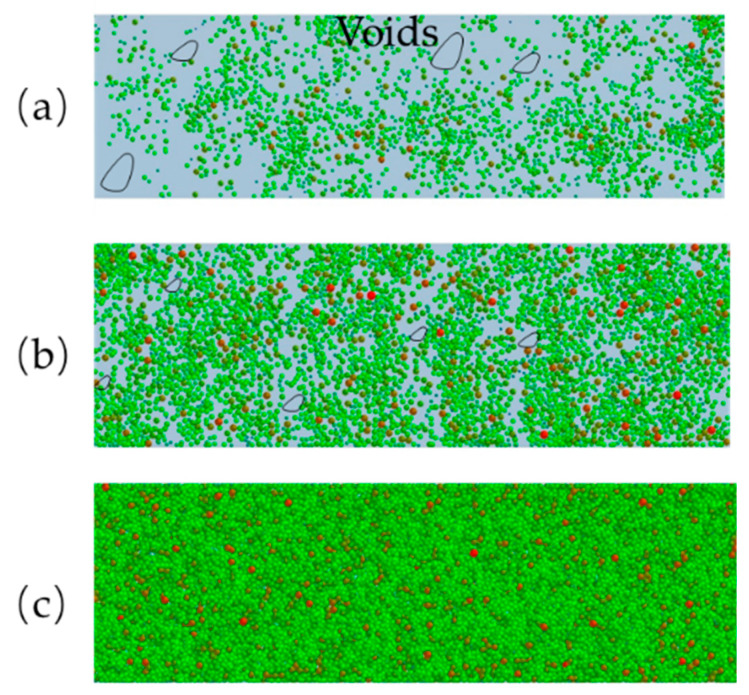
The powder-bed characteristics under different layer thickness values: (**a**) *H* = 50 μm; (**b**) *H* = 75 μm; and, (**c**) *H* = 150 μm.

When the layer thickness is 50 and 75 μm, the powder layer will have a lot of voids, as shown in Figure 17a,b. According to the literature [12], particle jamming becomes significantly stronger in the region before the blade when the layer thickness is too small. When the *H* is 50 and 75 μm, the particles are difficult to fall into the build platform due to particle jamming. Most of the particles are pushed out of the build platform by the roller. The powder-bed will have a lot of voids and this will affect the mechanical properties of the AM parts. When the powder layer thickness became smaller, the layer thickness has little effect on average fraction, as shown in the Figure 16a. However, when the layer thickness is greater than 150 μm, the powder-bed becomes more uniform and denser, as shown in the Figure 17c. This finding indicates that increasing layer thickness can obtain a good powder-bed compactness density and uniformity of powder-bed density.

From the perspective of powder-bed density, the layer thickness should be large. However, large layer thickness means that more particles need be bonded in the Inkjet 3D Printing process or melt in SLM processes, which leads to rough surface density and poor mechanical properties [36]. Roller-spreading is a key process in AM processes, and it needs to be related to the subsequent processing technology. Therefore, it is necessary to properly balance powder-bed density and the quality of AM parts.

## 4. Conclusions

In this work, DEM is used to simulate Al_2_O_3_ ceramic powder spreading by roller. The Hertz–Mindlin with JKR model of Al_2_O_3_ ceramic powder is established and calibrated. The powders are divided into three zones: I. Powder free-flow zone, II. Powder compression zone, and III. Powder layer zone. The effect of powder spreading parameters, including translational velocity *V_s_*, roller’s rotational speed *ω*, roller’ diameter *D,* and thickness of powder layer *H* are on Al_2_O_3_ ceramic powder-bed density are analyzed.

(1) The increased roller’s translational velocity lead to a reduced number of particles in powder layer zone. This will lead to poor powder-bed density. The roller’s rotational speed has little effect on the powder-bed density.
(2) When the roller’s diameter increases, more particles are in the powder compression zone. The normal contact force becomes strong, which enhances densification of powder-bed.
(3) Layer thickness is the most significant influencing factor on powder-bed density. When the layer thickness is 50 μm, most of the particles are pushed out of the build platform, forming a lot of voids. However, when the layer thickness is greater than 150 μm, the powder-bed becomes more uniform and denser.

The effect of roller-spreading parameters on the powder-bed density is studied in this paper. However, this paper does not optimize powder spreading parameters. In future work, an optimization model will be developed in order to improve the powder-bed density. The optimization model will be verified by experiments, which will provide a reliable basis for roller-spreading parameters optimization selection in AM.

## Figures and Tables

**Table 1:** List of equations used in the contact model.

Parameter	Equations
Normal stiffness constant	$S_{n}=2E^{*}\sqrt{R^{*}\delta_{n}}$
Damping ratio	$\beta =\frac{Ine}{\sqrt{In^{2}e+\pi^{2}}}$
Tangential stiffness	$S_{t}=8G^{*}\sqrt{R^{*}\delta_{n}}$
Equivalent shear modulus	$G^{*}={\left[\frac{\left(1-\gamma_{i}^{2}\right)}{G_{i}}+\frac{\left(1-\gamma_{j}^{2}\right)}{G_{j}}\right]}^{-1}$
Equivalent Young’s Modulus	$E^{*}={\left[\frac{\left(1-\gamma_{i}^{2}\right)}{E_{i}}+\frac{\left(1-\gamma_{j}^{2}\right)}{E_{j}}\right]}^{-1}$
Equivalent mass	$m^{*}={\left[\frac{1}{m_{i}}+\frac{1}{m_{j}}\right]}^{-1}$
Equivalent radius	$R^{*}={\left[\frac{1}{R_{i}}+\frac{1}{R_{j}}\right]}^{-1}$

**Table 2:** Discrete Element Method (DEM) parameters of Al_2_O_3_ ceramic particles.

Parameter	Value
Particle density (kg/m^3^)	3820
Particle size D (50) (μm)	48
Particle shear modulus (GPa)	3
Particle Poisson’s ratio	0.3
Roller density (kg/m^3^)	7800
Roller shear modulus (GPa)	80
Roller Poisson’s ratio	0.3
Static friction coefficient of particle-wall	0.20
Rolling friction coefficient of particle-wall	0.05
Restitution coefficient of particle-wall	0.52
Surface energy density of particle-wall (mJ/m^2^)	0.17
Static friction coefficient of particle-particle	0.34
Rolling friction coefficient of particle-particle	0.05
Restitution coefficient of particle-particle	0.50
Surface energy density of particle-particle (mJ/m^2^)	0.15

**Table 3:** The value of roller-spreading parameters.

Parameters	Value
Roller’s translational velocity *V_s_* (mm/s)	40/60/80/100/120/140/160
Roller’s rotational velocity *ω* (rpm)	40/80/120/160/200/240/280/320
Roller’s diameter *D* (mm)	3/4/5/6/7/8
Powder layer thickness *H* (μm)	50/75/100/125/150/175
